# Sperm Selection Using Cumulus Cell Column Improves Sperm DNA Integrity, Embryo Morphokinetics, and Clinical Outcomes Following ICSI: A Randomized Clinical Trial

**DOI:** 10.1002/rmb2.70000

**Published:** 2025-11-19

**Authors:** Mostafa Yousefi, Mohammad Ali Khalili, Maryam Eftekhar, Bryan J. Woodward, Fatemeh Anbari, Esmat Mangoli

**Affiliations:** ^1^ Research & Clinical Center for Infertility Yazd Reproductive Science Institute, Shahid Sadoughi University of Medical Science Yazd Iran; ^2^ X&Y Fertility Leicester UK

**Keywords:** cumulus cell column, embryo morphokinetics, ICSI, sperm selection

## Abstract

**Purpose:**

The selection of high‐quality spermatozoa affects embryo quality and, consequently, the success rates of clinical outcomes. This study aimed to evaluate the efficacy of a cumulus cell‐based sperm selection method for identifying high‐quality spermatozoa and to determine whether this process enhances ICSI clinical outcomes.

**Methods:**

A total of 88 ICSI cycles were analyzed, generating 640 embryos, 331 in the control group and 309 in the study group. Standard density gradient centrifugation was applied in the control group, while the study group underwent an additional selection step using cumulus cell columns (CCC) in microcapillary pipettes. Embryo development was monitored through time‐lapse imaging up to the blastocyst stage, and clinical outcomes were also recorded.

**Results:**

Results demonstrated a significant reduction in sperm DNA fragmentation following CCC selection (37.08% vs. 23.36%, *p* = 0.0001). Embryos derived from CCC‐selected sperm exhibited accelerated developmental kinetics and fewer cleavage abnormalities. Clinical outcomes were markedly enhanced in the study group, with higher implantation (58% vs. 28.4%), chemical pregnancy (81.8% vs. 50%), clinical pregnancy (77.3% vs. 25%), and live birth rates (72.7% vs. 25%) compared with controls (all *p* = 0.001).

**Conclusions:**

The Use of cumulus cell‐based sperm selection improves embryo quality and reproductive outcomes.

## 1 Introduction

Intracytoplasmic sperm injection (ICSI) involves the microinjection of a single sperm, selected based on morphology and motility, into the ooplasm [1]. Unlike conventional IVF, wherein sperm must traverse certain physiological barriers to fertilize the oocyte, ICSI bypasses these barriers entirely; this could increase the risk of selecting a genetically compromised sperm [2]. This could be attributed to the selection of sperm with compromised sperm DNA integrity, which may contribute to altered epigenetic and genetic outcomes in the offspring [3]. Incorporating functional criteria into sperm selection is critical to ensure that more healthy and mature sperm cells are selected for ICSI [4]. The selection of high‐quality sperm is a pivotal process in assisted reproductive technologies (ART), as it significantly influences the fertilization, embryo viability, and ultimately, the success rates of clinical outcomes [5].

Sperm plasma membrane integrity is crucial for the fertilization process, as it is associated with capacitation, oocyte binding, and the initiation of the acrosome reaction (AR). Consequently, numerous sperm selection methodologies have been proposed to exploit specific biophysical and biochemical properties of the sperm membrane to isolate high‐quality sperm with enhanced fertilization potential [6]. Hyaluronic acid (HA) constitutes the principal component of the extracellular matrix surrounding the cumulus–oocyte complex [7]. During the process of sperm maturation, elongating spermatids undergo extensive cytoplasmic extrusion and plasma membrane remodeling, resulting in the formation of HA‐binding sites [8]. In the context of fertilization, mature sperm interact with HA within the cumulus cell matrix via a specific sperm head protein known as PH20. This GPI‐anchored protein possesses hyaluronidase activity, which facilitates the AR and promotes penetration of the zona pellucida (ZP) [9]. The expression of HA‐binding receptors, including PH20, is restricted to sperm that have completed the full course of spermatogenesis and epididymal maturation. These receptors are critical for the acquisition of progressive motility and the ability to bind to both the ZP and the oolemma, thereby playing a pivotal role in successful fertilization [10].

Utilizing HA micro‐drops to aid sperm selection for ICSI has been explored. While some studies report improvements in fertilization, embryo development, and clinical pregnancy rates with HA‐selected sperm, findings remain inconsistent [11]. Meanwhile, the mechanism of using the cumulus cell column (CCC) is entirely different. The CCC mimics the natural environment of the oocyte, surrounded by cumulus cells. As sperm pass through this layer, the CCC acts as a biological filter, allowing only functionally competent sperm to pass, mimicking natural fertilization. In theory, CCCs help to select sperm with better motility, AR capability, and chromatin integrity [12].

An optimal sperm selection method should be non‐invasive, affordable, capable of accurately identifying high‐quality sperm, and should consistently yield high pregnancy and live birth rates. Although ICSI relies on the use of individual sperm for fertilization, there is still no universally accepted approach for selecting the most competent sperm. This study aimed to explore the efficacy of a modified sperm selection approach, based on the ability of cumulus cells to capacitate and hyperactivate sperm cells. Furthermore, this study looked to determine whether this technique can identify high‐quality sperm for successful clinical outcomes in ICSI.

## 2 Materials and Methods

### 2.1 Study Design

**FIGURE 1:**
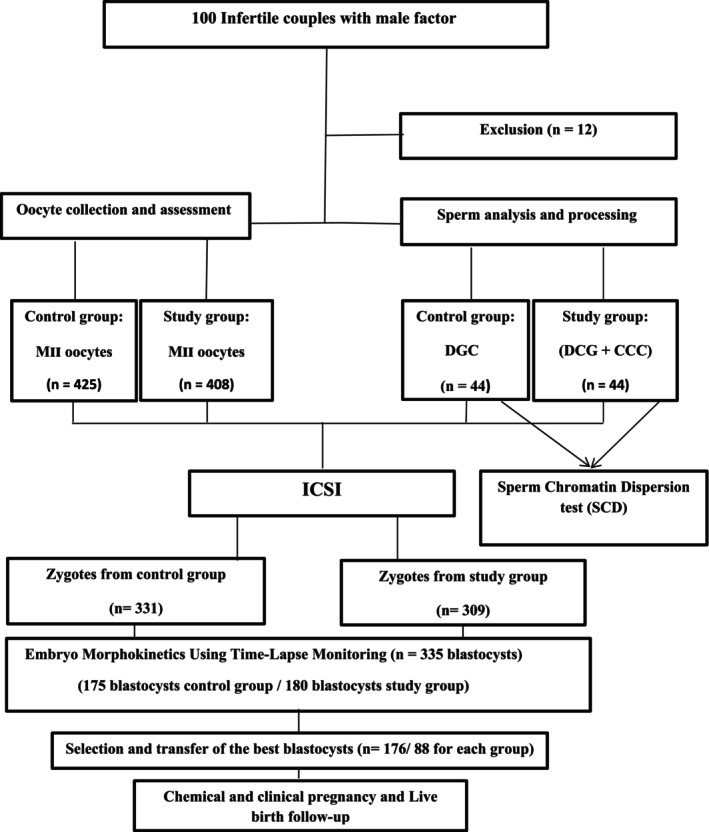
Schematic diagram of study design.

This study was approved by the Ethics Committee of the Research and Clinical Center for Infertility in Yazd, Iran (IR.SSU.MEDICINE.REC.1402.118). In this prospective study, 100 patients with male infertility were referred for ICSI treatment between October 2023 and November 2024. Informed consent was obtained to participate in this double‐blind randomized clinical trial (IRCTID: IRCT20180130038561N2). Moreover, this RCT was conducted and reported in accordance with the CONSORT (Consolidated Standards of Reporting Trials) reporting guidelines [13]. Patients were randomly assigned to either a control group (*n* = 44) or a study group (*n* = 44). Twelve cases were excluded from the study: four because sperm failed to pass through the CCC, and eight due to failing predefined criteria (flow chart, Figure 1). The block randomization method was performed both individually and using statistical software. The person performing the ICSI was blinded to which couples had been interviewed for the study and whether they agreed or declined to participate. In the control group, sperm samples were prepared, and selection was solely based on density gradient. In the study group, sperm selection involved density gradient plus passing through a cumulus cell column (CCC). A portion of the selected sperm in both groups underwent chromatin evaluation (SCD) testing. ICSI outcomes were then monitored and compared in vitro by tracking embryo development stages until blastocyst formation using a time‐lapse system, and in vivo by monitoring outcomes post embryo transfer, to pregnancy and live birth.

### 2.2 Inclusion and Exclusion Criteria

#### 2.2.1 Inclusion Criteria

(1) Male factor infertile couples, with males without a varicocele aged < 45 years, and females with regular cycles, normal vaginal ultrasound, aged < 38 years; (2) Estradiol level below 3000 (pg/mL) and presence of at least 3 follicles with size < 14 mm diameter on the trigger day; (3) A minimum of six metaphase II (MII) oocytes for ICSI.

#### 2.2.2 Exclusion Criteria

(1) Males with severe male factor according to WHO 2021 criteria, (2) HIV‐positive patients, (3) Females diagnosed with PCOS and/or endometriosis.

### 2.3 Semen Sample and Density Gradient Centrifugation

Fresh semen samples after 2 to 7 days of sexual abstinence were collected by masturbation on the day of oocyte retrieval in a sterile cup and placed in a 37°C incubator. Once liquefied, semen quality assessment was performed according to the WHO guidelines [14], and a two‐layered density gradient (40% and 80% Isolate) was used for sperm preparation.

### 2.4 Sperm Selection via the Cumulus Cells Column and Embryo Culture

Following oocyte collection, excess cumulus cells (CCs) were manually separated from mature oocytes using a pair of insulin needles. The CCs were collected into buffered culture medium and kept at 37°C until they were loaded into the capillary pipette. Oocytes were then treated with hyaluronidase and washed as per standard denudation prior to ICSI. The embryologist conducting ICSI was blinded to the sample allocations and group assignments.

**FIGURE 2:**
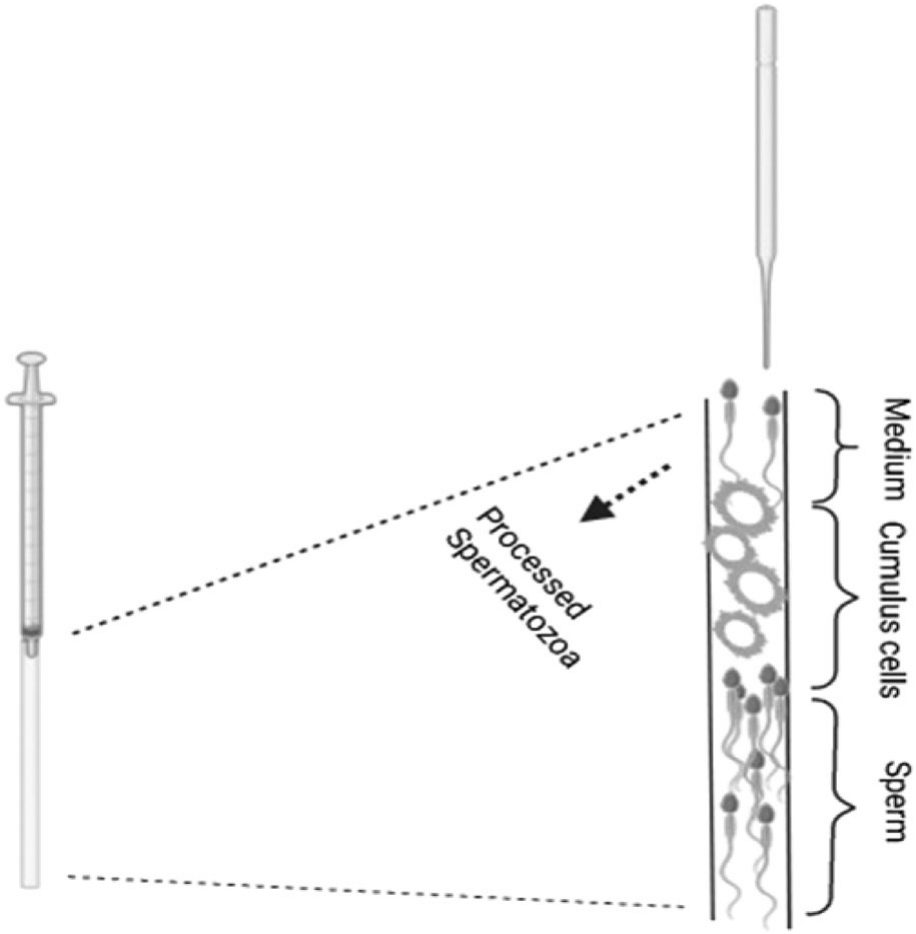
Schematic diagram of sperm selection using cumulus cells. The micro‐hematocrit capillary pipettes were filled with 2 cm of sperm medium, followed by 1 cm of collected CCs, and then 4 cm containing 1 million sperm cells. Each loaded capillary pipette, connected to an insulin syringe, was kept upright at room temperature for 45 min. After incubation, sperm that migrated through the CCs were collected at the top of the capillary.

For each couple, the respective female's CCs were used. Initially, 7 cm non‐heparinized micro‐hematocrit capillary pipettes (Sigma‐Aldrich) were rinsed with sterilized water. Each capillary pipette, connected to an insulin syringe, was then loaded in three layers. The bottom layer consisted of about 2 cm of sperm medium enriched with 10% human serum albumin (HSA; Albyrex 20, CSL Behring, Germany). Next, 1 cm of freshly collected cumulus cells was added to create a natural barrier that mimics the cumulus–oocyte complex in vivo. Finally, 4 cm of sperm containing roughly 1 × 10^6^ sperm cells was gently introduced above the cumulus layer. Each loaded capillary pipette, to maintain a sterile condition, was held upright under a laminar flow hood for 45 min. After incubation, sperm that had migrated through the CCs were extracted using a pulled Pasteur pipette inserted into the top of the capillary pipette (Figure 2). These selected sperm, considered to represent the most motile and physiologically competent population, were then used for the ICSI procedure. The collected sperm were then transferred directly to a PVP droplet in the ICSI dish. MII oocytes were then transferred to droplets in the same dish. ICSI was then performed, selecting sperm based on their morphology and motility. Following the fertilization check, fertilized oocytes were placed in 1‐step medium (Origio, Denmark) and incubated in a time‐lapse system until day 5. Embryo quality was evaluated by an embryologist blinded to the group allocations. Based on this assessment, the number of top‐quality embryos and their transfer order were determined. At this stage, the gynecologist and the couple were informed, and the number of embryos to be transferred (one or two) was confirmed. In cases where a fresh embryo transfer was canceled, the embryos were vitrified.

### 2.5 Determination of Sperm Chromatin Integrity

Sperm DNA integrity was assessed using the Sperm Chromatin Dispersion (SCD) test. Sperm cells were classified based on halo size around the nucleus, indicating DNA fragmentation. Large or medium halos represented intact DNA, while small, absent, or degraded halos indicated fragmentation. A total of 200 spermatozoa per sample were analyzed, and the percentage of fragmented DNA was calculated. The assay worked on the principle that intact DNA dispersed chromatin (forming halos), whereas fragmented DNA showed minimal or no dispersion [15].

### 2.6 Cleavage Abnormality

Based on their cleavage patterns, embryos were classified into five categories: (I) fragmentation—presence of small, incomplete cell‐like fragments; (II) multinucleation—the occurrence of multiple nuclei within a single cell at the 2‐ or 4‐cell stage; (III) uneven cleavage—blastomeres of unequal size; (IV) reverse cleavage—when, before reaching the 8‐cell stage, two separate cells fuse into one or the cytoplasm contracts without dividing; and (V) direct cleavage—a single cell dividing directly into three or more cells [16].

### 2.7 Morphology and Morphokinetic Parameters

The fertilization rate was determined by dividing the number of 2PNs by the number of mature (MII) oocytes. Zygotes were loaded in a 9‐well Primo Vision culture dish (Vitrolife, Denmark) and incubated in a Primo Vision time‐lapse imaging (TLI)system at 37°C with 6% CO_2_ and 5% O_2_ for 5 days. The assessed time points included pronuclear fading (tPNf), progression from the 2‐cell to 8‐cell stages (t2 to t8), start of compaction (tSC), completion of compaction or time to Morula formation (tM), start of blastulation (tSB), and time to blastocyst formation (tB). Additionally, calculated intervals included: the duration from 2‐cell to 4‐cell stage (CC2 = t4–t2), from 4‐cell to 8‐cell stage (CC3 = t8–t4), from PN fading to the 2‐cell stage (S1 = t2–tPNf), from 3‐cell to 4‐cell stage (S2 = t4–t3), and from 5‐cell to 8‐cell stage (S3 = t8–t5) [15].

Embryo development was monitored using TLI, with frames captured every 10 min across 7 focal planes. The blastocysts were considered high quality if they were fully expanded with normal inner cell mass (ICM) and trophectoderm structures. Blastocysts were categorized into six stages based on the extent of blastocyst cavity expansion. The quality of the ICM or trophectoderm was graded as A, B, or C, depending on the number of cells present. Blastocysts with a score of ≥ 3BB were classified as high‐quality, whereas those with either an ICM or trophectoderm score of ≥ B were deemed available [17]. Two of the best‐quality embryos were selected based on morphology and morphokinetics and transferred on day 5, in line with our center's policy. Chemical pregnancy was confirmed by measuring β‐hCG levels, while the presence of a gestational sac and fetal heartbeat identified clinical pregnancy. The implantation rate was calculated as the number of gestational sacs divided by the total number of embryos transferred within each group [1].

### 2.8 Statistical Analysis

Data were presented as mean ± SD. The Shapiro–Wilk test was used to assess the normal distribution of the data. After evaluating the normal distribution of the data, one of the *T*‐Test or Mann–Whitney tests was used to compare the data between the two groups based on whether they were normal or not. The *χ* ^2^ test was used to compare qualitative data. *p* < 0.05 was considered significant.

## 3 Results

**FIGURE 3:**
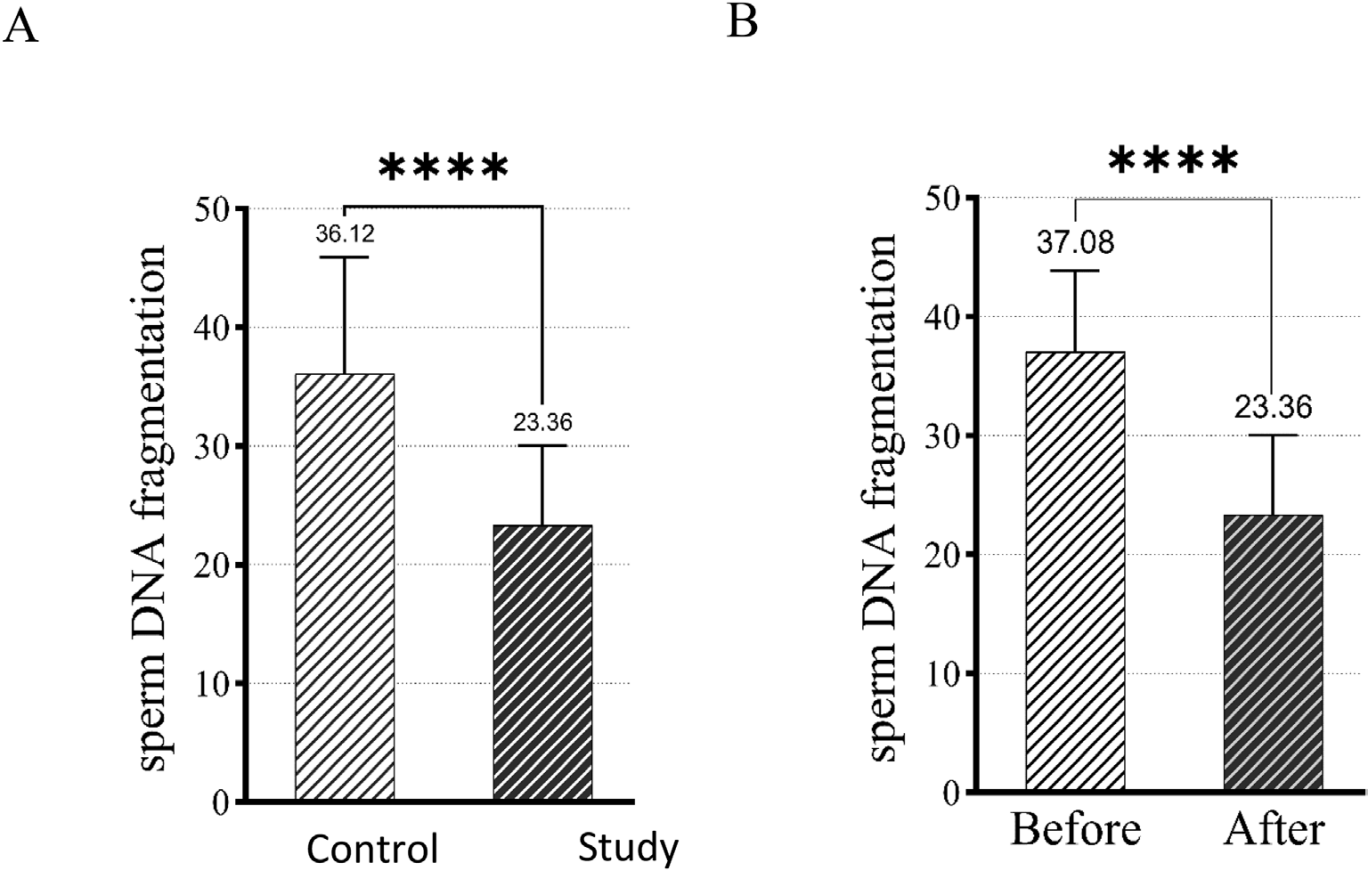
Sperm Chromatin Dispersion (SCD) test. (A) Comparison of sperm DNA fragmentation between the control and study groups using the SCD test. A significantly lower percentage of sperm with chromatin dispersion was observed in the study group (*p* = 0.0001). (B) Bar graph illustrating sperm DNA fragmentation rates before and after selection using the CCC in the study group. A significantly lower percentage of sperm with chromatin dispersion was observed (*p* = 0.0001).

A total of 44 couples were allocated to the study group (yielding 309 embryos) and another 44 to the control group (yielding 331 embryos), with 88 embryos transferred in each group at a rate of two embryos per cycle. The first participant was enrolled on November 27, 2023. The characteristics of the couples and semen parameters were comparable between the groups (Table 1). Embryo culture outcomes are presented in Table 2. The mean fertilization rate was higher in the control compared to the study group (77.88% vs. 75.73%, *p* = 0.05). Additionally, the blastocyst formation rate was comparable between the groups (52.9% vs. 58.3%, *p* = 0.17). Moreover, all blastocysts obtained from both the control and study groups were classified as good quality [18]. Embryonic development in the study group was significantly faster than that in the controls, with earlier onset in all morphokinetic parameters (*p* < 0.05; Table 3). The rate of cleavage abnormalities was significantly higher in the control than in the study group (Table 4). The information on morphokinetic parameters and cleavage abnormalities of transferred embryos is presented in Tables 5 and 6. The SCD results showed a significant reduction in DNA fragmentation between the control and study groups, respectively (36.12% vs. 23.36%, *p* = 0.0001), as well as before and after sperm passage through the CCC (37.08% vs. 23.36%, *p* = 0.0001) (Figure 3).

**TABLE 1:** The basic lab characteristics of patients in different groups.

Characteristics	Control group	Study group	*p*
	Mean ± SD (median)	Mean ± SD (median)	
Female age (years)	35.25 ± 2.50 (35.50)	33.29 ± 3.76 (34.00)	0.45
Male age (years)	37.75 ± 2.06 (38.00)	35.42 ± 4.53 (35.50)	0.47
Duration of Infertility (years)	8.25 ± 5.73 (7.00)	6.33 ± 4.16 (5.00)	0.66
Estradiol (pg/mL)	2362.50 ± 1001.97 (1950.00)	2165.47 ± 907.47 (1990.00)	0.83
AMH (ng/mL)	1.75 ± 0.59 (1.70)	2.59 ± 1.93 (2.00)	0.89
No. of oocytes retrieved (*n*)	11.75 ± 3.09 (11.00)	11.84 ± 4.58 (11.00)	0.75
No. of mature oocytes, (*n*)	9.00 ± 0.81 (9.00)	9.45 ± 3.64 (8.50)	0.32
Sperm concentration × 10^6^	41.25 ± 21.74 (42.50)	28.92 ± 15.92 (25.00)	0.06
Prog (%)	30.00 ± 12.24 (32.50)	27.29 ± 13.82 (25.00)	0.60
Non prog (%)	14.50 ± 6.40 (15.00)	14.68 ± 10.25 (10.50)	0.76
Immotile (%)	55.50 ± 18.19 (53.50)	58.03 ± 16.11 (60.00)	0.30
Normal morphology (%)	2.50 ± 0.57 (2.50)	2.16 ± 0.75 (2.00)	0.67

Abbreviations: Non‐prog, non‐progressive motility; Prog, progressive motility.

**TABLE 2:** Comparison of laboratory characteristics between the two groups.

Variables		Control	Study	*p*
No. of oocytes		514	508	—
No. of MII oocytes		425	408	
No. of embryos (2PN)		331 (77.88%)	309 (75.73%)	
No. of embryos (blastocyst)		175 (52.9%)	180 (58.3%)	
Blastocyst grading rate %, (*n*)	4AA	10.9 (19/175)	69.4 (125/180)	0.001*
	4BB	59.4 (104/175)	25.6 (46/180)	0.001*
	3AA	22.9 (40/175)	4.4 (8/180)	0.001*
	3BB	6.8 (12/175)	0.6 (1/180)	0.001*

Abbreviation: PN, pronuclei.

* *p* < 0.05 was significant.

**TABLE 3:** Comparison of morphokinetic parameters between all embryos in the two groups.

Parameters (*h*)	Control (*n* = 331)	Study (*n* = 309)	*p*
	Mean ± SD (median)	Mean ± SD (median)	
tPNf	28.35 ± 1.62 (28.00)	22.54 ± 1.93 (22.00)	0.001
t2	33.67 ± 3.01 (34.00)	25.15 ± 2.71 (25.00)	0.001
t3	40.45 ± 3.25 (41.00)	27.77 ± 3.57 (27.00)	0.001
t4	47.69 ± 5.03 (49.00)	32.56 ± 4.26 (31.00)	0.001
t5	55.76 ± 6.64 (57.50)	36.83 ± 4.45 (35.00)	0.001
t6	64.35 ± 8.63 (67.00)	40.39 ± 4.75 (39.00)	0.001
t7	71.55 ± 9.85 (75.00)	44.16 ± 4.77 (43.00)	0.001
t8	80.10 ± 11.05 (85.00)	48.72 ± 4.80 (48.00)	0.001
t2 to t8	46.44 ± 9.90 (50.00)	23.38 ± 3.64 (23.00)	0.001
S1	5.33 ± 2.66 (5.00)	2.51 ± 1.58 (2.00)	0.001
S2	7.21 ± 2.70 (7.00)	4.70 ± 1.55 (4.00)	0.001
S3	24.28 ± 6.24 (26.00)	11.94 ± 2.72 (12.00)	0.001
CC2	14.12 ± 3.70 (15.00)	7.39 ± 2.39 (7.00)	0.001
CC3	32.40 ± 7.68 (35.00)	16.11 ± 3.01 (16.00)	0.001
tSC	92.83 ± 6.57 (95.00)	72.54 ± 5.67 (72.00)	0.001
tM	103.63 ± 6.16 (106.00)	90.19 ± 4.53 (89.00)	0.001
tSB	107.65 ± 5.29 (110.00)	95.23 ± 4.59 (95.00)	0.001
tB	120.47 ± 2.78 (120.00)	114.99 ± 3.22 (115.00)	0.001

*Note:* Values are expressed as mean ± SD. CC2 = t4–t2; CC3 = t8–t4; S1 = t2–tPNf; S2 = t4–t3; S3 = t8–t5.

Abbreviations: t2 to t8, two to eight discrete cells; tB, time to blastocyst formation; tM, time to Morula formation; tPNf, time of pronuclear fading; tSB, start of blastulation; tSC, start of compaction.

**TABLE 4:** Comparison of embryo cleavage abnormalities between all embryos in two groups.

Cleavage pattern %, (*n*)	Control (*n* = 331)	Study (*n* = 309)	*p*
Fragmentation	95.2 (315/331)	58.3 (180/309)	0.001*
Multinucleation	26.6 (88/331)	12.0 (37/309)	0.001*
Uneven	47.1 (156/331)	20.4 (63/309)	0.001*
Reverse	49.8 (165/331)	19.7 (61/309)	0.001*
Direct	22.7 (75/331)	15.5 (48/309)	0.02*
Vacuole	28.2 (93/331)	21.4 (66/309)	0.04*

*Note:* Values are expressed as a percentage.

* *p* < 0.05 was significant.

**TABLE 5:** Comparison of morphokinetic parameters between transferred embryos in two groups.

Parameters (*h*)	Control (*n* = 88)	Study (*n* = 88)	*p*
	Mean ± SD (median)	Mean ± SD (median)	
tPNf	28.25 ± 1.58 (29.00)	26.63 ± 2.58 (27.00)	0.001*
t2	33.38 ± 4.27 (32.00)	31.63 ± 2.43 (31.00)	0.24
t3	38.25 ± 4.74 (38.00)	36.72 ± 3.14 (37.00)	0.08
t4	44.00 ± 5.04 (44.00)	41.84 ± 3.68 (43.00)	0.74
t5	50.25 ± 3.73 (49.50)	46.82 ± 4.51 (48.00)	0.001*
t6	55.38 ± 3.62 (54.50)	51.24 ± 4.94 (52.50)	0.001*
t7	62.38 ± 3.81 (60.50)	55.58 ± 5.11 (57.00)	0.001*
t8	69.13 ± 4.88 (67.00)	60.20 ± 5.52 (62.00)	0.001*
t2 to t8	35.75 ± 2.81 (35.00)	28.66 ± 5.20 (30.00)	0.001*
S1	5.13 ± 3.64 (3.50)	4.99 ± 2.99 (4.00)	0.01*
S2	5.75 ± 1.75 (5.50)	5.12 ± 2.14 (5.00)	0.08
S3	18.88 ± 2.85 (19.50)	13.47 ± 4.15 (14.00)	0.10
CC2	10.63 ± 2.50 (11.00)	10.05 ± 3.23 (9.50)	0.91
CC3	25.13 ± 2.80 (25.50)	18.29 ± 4.51 (18.00)	0.002*
tSC	83.00 ± 5.70 (82.50)	76.37 ± 7.21 (74.00)	0.001*
tM	101.25 ± 3.45 (100.00)	95.04 ± 4.93 (95.00)	0.001*
tSB	108.25 ± 4.43 (108.00)	98.55 ± 13.54 (99.00)	0.001*
tB	124.50 ± 3.25 (125.00)	117.92 ± 3.81 (118.00)	0.001*

*Note:* Values are expressed as mean ± SD. CC2 = t4–t2; CC3 = t8–t4; S1 = t2–tPNf; S2 = t4–t3; S3 = t8–t5.

Abbreviations: t2 to t8, two to eight discreet cells; tB, time to blastocyst formation; tM, time to Morula formation; tPNf, time of pronuclear fading; tSB, start of blastulation; tSC, start of compaction.

* *p* < 0.05 was significant.

**TABLE 6:** Comparison of embryo cleavage abnormalities between transferred embryos in the two groups.

Cleavage pattern %, (*n*)	Control	Study	*p*
No. of embryos	88	88	
Fragmentation	62.5 (55/88)	50.0 (44/88)	0.09
Multinucleation	3.4 (3/88)	10.2 (9/88)	0.06
Uneven	35.2 (31/88)	15.9 (14/88)	0.001*
Reverse	28.4 (25/88)	14.8 (13/88)	0.06
Direct	8.0 (7/88)	6.8 (6/88)	0.04*
Vacuole	12.5 (11/88)	6.8 (6/88)	0.20

*Note:* Values are expressed as a percentage.

* *p* < 0.05 was significant.

### 3.1 Clinical Outcomes

The rates of implantation and chemical pregnancy were significantly higher, while the clinical pregnancy and live birth rates were approximately threefold, in the study group compared to the control (*p* < 0.05; Table 7). In the control group, 11 sacs were observed without fetal heart activity, while in the study group, this was the case for 2 sacs, resulting in clinical pregnancy rates of 25.0% and 77.3% for the control and study groups, respectively. The live birth rates in the control and study groups were 25.0% and 72.7%, respectively. Also, 60 live births were recorded, including 17 twin pregnancies. Additionally, there were 4 miscarriages (2 in each group) and 1 case of ectopic pregnancy in the study group. In terms of neonatal sex distribution, the control group resulted in 11 male and 3 female births, while the study group resulted in 42 male and 4 female births.

**TABLE 7:** Clinical outcomes between the transferred embryos in two groups.

Variables %, (*n*)		Control	Study	*p*
Implantation		28.4 (25/88)	58 (51/88)	0.001*
Chemical pregnancy		50 (22/44)	81.8 (36/44)	0.001*
Clinical pregnancy		25.0 (11/44)	77.3 (34/44)	0.001*
Live birth		25.0 (11/44)	72.7 (32/44)	0.001*
Abortion		18 (2/11)	5 (2/34)	
EP		0.0 (0/11)	2 (1/34)	
Gender	Male	78.6 (11/14)	91.3 (42/46)	0.33
	Female	21.4 (3/14)	8.7 (4/46)	

Abbreviation: EP, ectopic pregnancy.

* *p* < 0.05 was significant.

## 4 Discussion

We utilized the sperm penetration ability through the CCC to select mature sperm for ICSI. In this method, the three‐dimensional structure of the cumulus oophorus was preserved within a glass capillary. Considering the small inner diameter of the capillary pipette, the cumulus matrix's elasticity allowed the cell mass to fill the capillary's lumen. This configuration ensured that only sperm that had successfully penetrated the cumulus oophorus accumulated in the medium above it. The CCC acted as a biological filter, recreating important physical and chemical guidance mechanisms, such as rheotaxis and chemotaxis, found in the natural female reproductive tract. This helped in selecting sperm with optimal fertilization potential, mimicking the natural selection process. The number of sperm recovered using this method depended on both the length of the CCC and the incubation period. We selected a 45‐min incubation time and a 1 cm CCC length as the baseline conditions. This duration allowed for optimal sperm penetration, ensuring adequate interaction between the sperm and the cumulus matrix. To the best of our knowledge, this is the first study to investigate the developmental stages of embryos derived from sperm selection using this technique and monitored via a TLI system. Our findings indicated that the incidence of high‐quality blastocysts was significantly higher in the study group compared to the controls. Moreover, there was a notable reduction in DNA fragmentation and cleavage abnormalities, along with significant improvements in rates of implantation, pregnancy, and live birth.

Mature sperm that exhibit the ability to bind HA are typically characterized by normal morphology, low levels of nuclear DNA fragmentation, efficient ZP binding capacity, intact DNA with appropriate chromatin packaging, successful cytoplasmic extrusion, and reduced rates of aneuploidy [19]. These cells also retain the capacity to undergo key physiological processes, including the AR and hyperactivated motility [20, 21]. Sperm expressing functional HA receptors and exhibiting robust hyaluronidase activity demonstrate an enhanced capacity to penetrate the extracellular matrix, adhere to the ZP, and improve blastocyst formation [9, 22]. Additionally, these mature sperm display an improved responsiveness to progesterone secreted by cumulus cells, which contributes to enhanced embryo quality [23]. In contrast, sperm lacking HA‐binding sites often display elevated levels of cytoplasmic enzymes, such as creatine kinase, and increased markers of oxidative stress, including lipid peroxidation and DNA fragmentation. These aberrations are frequently associated with defective spermatogenesis, meiotic errors, and elevated rates of aneuploidy [24]. Notably, HA‐bound spermatozoa have been shown to exhibit a 5.4‐fold reduction in chromosomal aneuploidy compared to their non‐HA‐bound counterparts, underscoring the utility of HA binding as a marker of sperm maturity and genomic integrity [25].

Cumulus cells play an essential role in helping only the best sperm reach and fertilize the oocyte. They secrete and interact with various factors that influence sperm function. Hyaluronic acid, a key part of the cumulus matrix, forms a gentle barrier that slows down immature sperm and lets only the most capable ones pass through. Progesterone, produced by these cells, triggers important changes in the sperm—raising calcium levels, inducing the acrosome reaction, and increasing motility—so they're better prepared to fertilize the oocyte. Progesterone activates the CatSper channel by interacting with the enzyme ABHD2 (α/β hydrolase domain–containing protein 2) [26, 27]. Under normal conditions, the human CatSper channel is kept inactive by the endocannabinoid 2‐arachidonoylglycerol (2‐AG). When progesterone binds to ABHD2, the enzyme breaks down 2‐AG, which removes this inhibition and allows CatSper to open. The resulting influx of calcium ions triggered by progesterone plays a key role in sperm functions such as capacitation, chemotaxis, hyperactivation, and the acrosome reaction [28, 29]. Other small molecules, such as ATP, cAMP, and nitric oxide, fine‐tune sperm metabolism and signaling, improving their readiness for fertilization. At the same time, antioxidants like glutathione protect sperm from oxidative damage, and chemoattractants like prostaglandins and CXCL12 help guide them toward the oocyte [30]. Cumulus cells also “clean up” the sperm by removing excess cholesterol, immature surface proteins, and harmful reactive oxygen species, all of which support proper capacitation and DNA protection. Altogether, these cells act like a smart biological filter—selecting, protecting, and fine‐tuning sperm to maximize the chances of successful fertilization [27].

Hong et al. were the first to propose the use of a CCC as a method for selecting high‐quality sperm [31]. They reported that sperm capable of traversing the cumulus oophorus exhibited superior morphological characteristics, enhanced ZP binding capacity, and distinct motility patterns [31]. While they utilized a Pasteur pipette for the CCC setup, allowing potential escape routes for sperm around the cumulus oophorus, we employed a hematocrit tube with a narrower internal diameter. This modification was designed to minimize the possibility of sperm bypassing the cumulus structure, thereby forcing them to actively traverse the cumulus oophorus. As a result, it potentially enhances the physiological relevance and stringency of the selection process.

Subsequently, the utility of the CCC in selecting motile sperm was further supported by Naknam and associates [20]. They observed a reduced rate of DNA fragmentation in the selected sperm population [20], which is consistent with our findings. Several factors could explain the role of sperm DNA fragmentation (SDF) in ART outcomes. First, an increase in SDF can significantly delay key embryo morphokinetic parameters that are strongly linked to successful blastocyst development [32]. Second, if the level of DNA damage surpasses the oocyte's capacity for repair, it may result in incomplete repair of sperm DNA, leading to impaired embryo development [33]. Third, high SDF can interfere with sperm chromatin decondensation, which increases the risk of chromosomal abnormalities [34].

In 2018, Wang and colleagues modified the technique by incorporating horizontal CCC into ICSI culture dishes using sibling oocytes [35]. Their findings revealed that sperm penetrating the cumulus cells resulted in increased fertilization rates and the production of top‐quality blastocysts. However, insignificant differences were observed in implantation or pregnancy rates when compared with the conventional method [35].

According to Sabet et al. [1], selecting sperm via a CCC significantly improved cleavage embryo quality and pregnancy outcomes, consistent with our data. Furthermore, results from TUNEL and CMA3 assays indicated a marked reduction in DNA fragmentation and protamine deficiency in the selected sperm population. These findings were consistent with those reported by Wang et al., suggesting that enhanced fertilization outcomes may be attributed to the selective interaction with cumulus cells, which permits the penetration of functionally competent sperm [35]. The turning point of the present study was to fully record the morphokinetics of the blastocyst stage via TLI. The early embryo's initial three divisions rely mostly on maternal transcripts, while the paternal genome starts to play a role only after the 4–8 cell stage, which aligns with the activation of the embryonic genome [5].

Embryo development in the study group was significantly faster than in the control, with an earlier onset in all morphokinetic parameters. It is well recognized that each time point signifies a stage in embryonic development progression, and any disruption in this process may indicate an abnormality in embryonic development. Pronuclear fading (tPNf) indicates successful fertilization and syngamy. Deviations from the expected timing of tPNf may indicate early developmental arrest, associated with chromosomal segregation errors. Moreover, an accelerated timing of tPNf may reflect enhanced embryonic competence, often associated with improved developmental potential and higher implantation rates [36, 37].

The transition to the 2‐cell stage (t2), representing the first mitotic division, is positively correlated with developmental competence; aberrant timing at this stage has been linked to reduced embryo quality. The earlier transition to the 2‐cell stage is associated with improved embryo quality and successful implantation [4, 38]. The subsequent cleavage stage is essential for assessing cleavage synchrony, a strong predictor of embryo viability. Asynchronous cleavage at these stages may reflect the underlying chromosomal abnormalities [39, 40].

The timing of compaction, which marks the transition to the morula stage (tM), plays a crucial role in subsequent blastocyst formation. Delays in compaction have been linked to reduced implantation potential [41]. Conversely, earlier compaction has been associated with improved embryo quality, higher blastocyst formation, and implantation outcomes [42]. Similarly, the formation of the blastocyst (tB) at the appropriate developmental time is essential, as delayed blastulation may indicate underlying metabolic stress or chromosomal abnormalities, such as aneuploidy [43]. Beyond absolute time points, the temporal intervals between cleavage events provide important insights into embryo quality, and synchrony metrics reflect the consistency of cell division [44]. A pattern was evident in the embryos obtained from the study group and the corresponding transfer outcomes.

Moreover, the rate of DNA fragmentation and cleavage abnormalities was significantly lower in the study group. Additionally, the study group maintained a high pregnancy conversion rate from chemical to clinical pregnancy. This suggests that the intervention may positively affect early pregnancy maintenance, thereby reducing early pregnancy loss. These improvements are likely due to the technique's ability to select physiologically superior or more functionally competent sperm through an integrated selection process. This approach may involve the sperm's capacity to traverse HA secreted by cumulus cells, an indicator linked to greater sperm maturity, and responsiveness to cumulus‐derived progesterone. Overall, improvements in blastocyst quality and outcomes can be attributed to selecting higher‐quality sperm.

## 5 Conclusion

The use of cumulus cell‐based sperm selection resulted in significantly higher implantation and chemical pregnancy rates, as well as approximately threefold increases in clinical pregnancy and live birth rates. Using this method, sperm cells with key physiological processes in the fertilization cascade can be selected without the risks associated with cytoplasmic injection. Additional studies with a larger sample size and single‐embryo transfer are needed to validate our findings.

## Conflicts of Interest

The authors declare no conflicts of interest.

## Data Availability

The data that support the findings of this study are available from the corresponding author upon reasonable request.
