# Correction to “Sperm Selection Using Cumulus Cell Column Improves Sperm DNA Integrity, Embryo Morphokinetics, and Clinical Outcomes Following ICSI: A Randomized Clinical Trial”

**DOI:** 10.1002/rmb2.70019

**Published:** 2026-01-27

M. Yousefi, M. A. Khalili, M. Eftekhar, B. J. Woodward, F. Anbari, and E. Mangoli, “Sperm Selection Using Cumulus Cell Column Improves Sperm DNA Integrity, Embryo Morphokinetics, and Clinical Outcomes Following ICSI: A Randomized Clinical Trial,” *Reproductive Medicine and Biology* 24, no. 1 (2025): e70000, https://doi.org/10.1002/rmb2.70000.

In Table 7 on page 8, the values in Row 5, Column 2 (Abortion in the control group) and Row 6, Columns 2 and 3 (EP in the control and study groups) were incorrect. The correct value for Row 5 is 0.0 (0/11). For Row 6, the correct values are 0.0 (0/22) in the control group and 2.7 (1/36) in the study group.

**TABLE 7:** Clinical outcomes between the transferred embryos in two groups.

Variables% (*n*)	Control (*n* = 44)	Study (*n* = 44)	*p*
Implantation	28.4 (25/88)	58 (51/88)	0.001*
Chemical pregnancy	50 (22/44)	81.8 (36/44)	0.001*
Clinical pregnancy	25.0 (11/44)	77.3 (34/44)	0.001*
Live birth	25.0 (11/44)	72.7 (32/44)	0.001*
Abortion	0.0 (0/11)	5 (2/34)	
EP^a^	0.0 (0/22)	2.7 (1/36)	
Gender			
Male	78.6 (11/14)	91.3 (42/46)	0.33
Female	21.4 (3/14)	8.7 (4/46)	

Abbreviation: EP, ectopic pregnancy.

* *p* < 0.05 was significant.

^a^ Ectopic pregnancy rate is calculated per chemical pregnancy.

As a result, under 3.1|Clinical Outcomes on page 5, the sentence “Additionally, there were 4 miscarriages (2 in each group) and 1 case of ectopic pregnancy in the study group.” should be corrected to “In the study group, there were 2 miscarriages and 1 ectopic pregnancy.”

We apologize for these errors.

